# Supplementary material for: Canonical BMP Signaling Executes Epithelial-Mesenchymal Transition Downstream of SNAIL1
Source: Cancers (Basel). 2020 Apr 21;12(4):1019. doi: 10.3390/cancers12041019 (PMC7226241; doi:10.3390/cancers12041019)
Supplement: Supplementary file 1 [file cancers-12-01019-s001.zip › cancers-778887-suppl-figures.pdf]

Canonical BMP Signaling Executes Epithelial-Mesenchymal Transition Downstream of SNAIL1

Patrick Frey, Antoine Devisme, Monika Schrempp, Geoffroy Andrieux, Melanie Boerries and Andreas Hecht

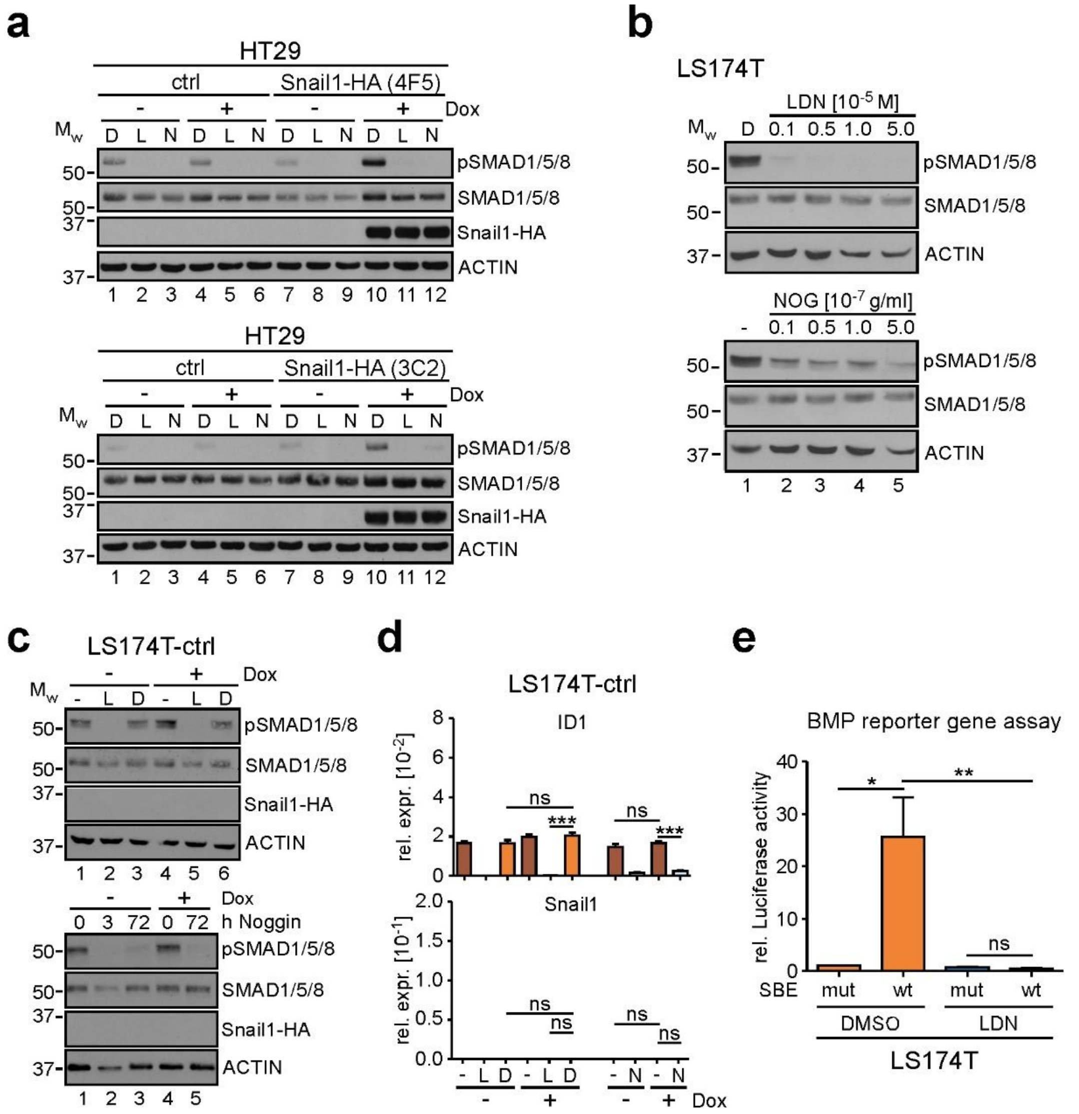

Figure S1. Analyses of BMP pathway **a**ctivity in LS174T and HT29 cells and efficacy of BMP pathway inhibitors. (**a**) Western Blot analyses of whole-cell lysates from HT29-control (ctrl) and HT29-Snail1-HA cells (cell clones 4F5 and 3C2). Cells had been left untreated or had received 1 µg ml^{-1} Dox and DMSO (D), 50 nM LDN193189 (L), or 100 ng ml^{-1} Noggin (N) for 72 h prior to harvest. Names of detected proteins are indicated on the right. Positions of molecular weight (M$_W$) standards in kDa are given on the left. Detection of ACTIN was used as control for equal loading. (**b**) Western Blot analyses of whole-cell lysates from LS174T cells. Names of detected proteins are indicated on the right. LS174T cells had been left untreated (-), treated with DMSO (D), or the indicated concentrations of LDN193189 (LDN) or Noggin (NOG) for 1 h prior to harvest. Positions of molecular weight (M$_W$) standards in kDa are given on the left. Detection of ACTIN was used as control for equal loading. (**c**) Western Blot analyses of whole-cell lysates from LS174T-control (ctrl) cells. Names of detected proteins are indicated on the right. LS174T-ctrl cells had been left untreated or treated with 0.1 µg ml^{-1} Dox and DMSO (D, 72 h), 50 nM LDN193189 (L, 72 h), or 100 ng ml^{-1} Noggin for the indicated time spans prior to harvest. Positions of molecular weight (M$_W$) standards in kDa are given on the left. Detection of ACTIN was used as control for equal loading. (**d**) qRT-PCR analyses of mRNA expression in LS174T-ctrl cells. Where indicated, cells were treated with 0.1 µg

ml^{-1} Dox and DMSO (D), 50 nM LDN193189 (L), or 100 ng ml^{-1} Noggin (N) for 72 h. Shown is the mean+SEM; n = 3. Rel. expr.: relative expression normalized to that of *GAPDH*. ns: not significant. ***: $p < 0.001$. (**e**) Luciferase reporter gene assay in LS174T cells using a BMP signaling reporter plasmid. Cells were treated with DMSO or 50 nM LDN193189 (LDN) for 48 h. Luciferase activity was normalized to the mutated construct under DMSO treatment. For the comparison of mut with wt under DMSO treatment, significance was therefore determined using two-tailed one sample t test. Shown is the mean + SEM, n = 7. mut: mutated, rel.: relative, SBE: SMAD-binding element, wt: wild-type. ns: not significant. *: $p < 0.05$, **: $p < 0.01$.

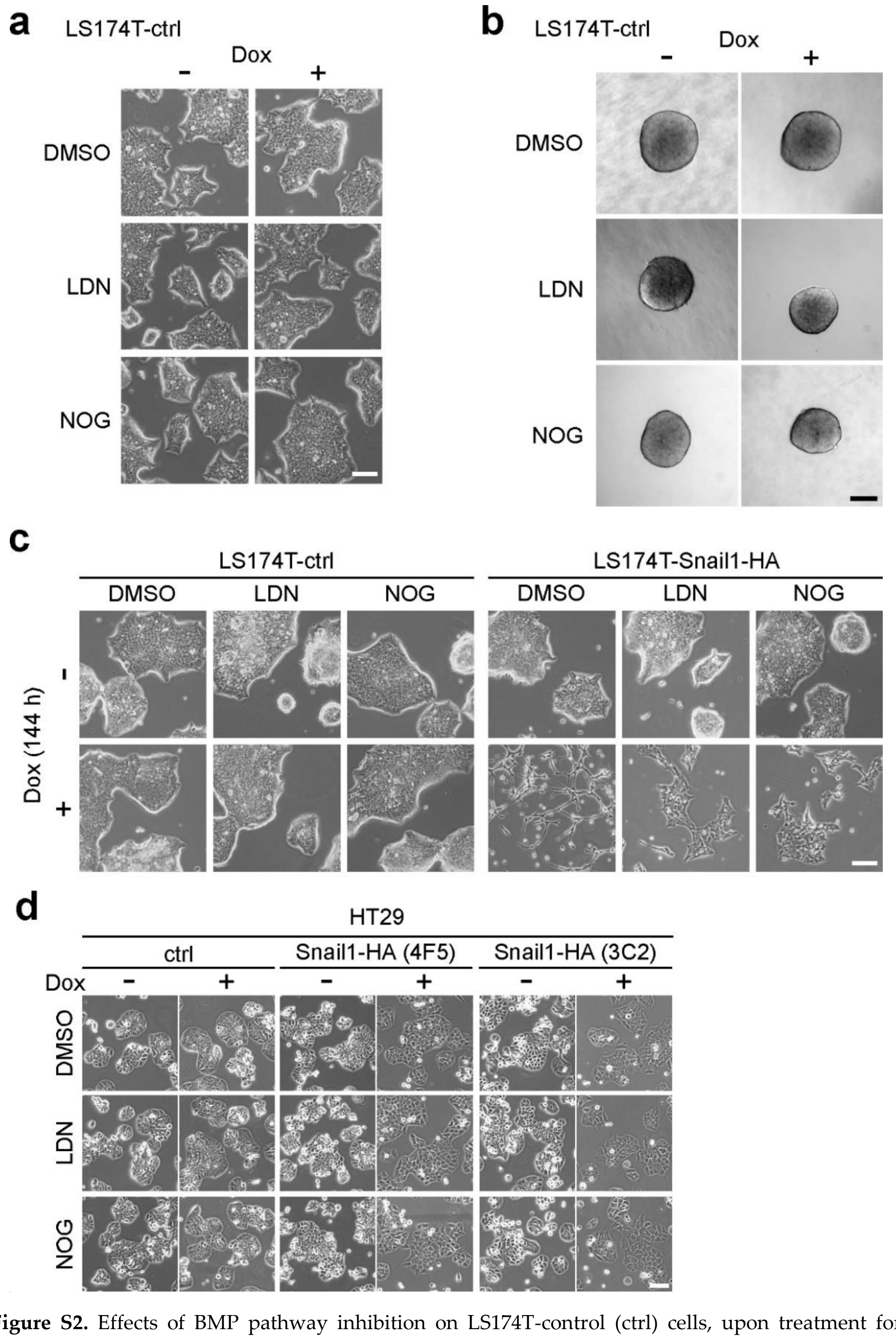

Figure S2. Effects of BMP pathway inhibition on LS174T-control (ctrl) cells, upon treatment for longer periods of time, and on HT29-ctrl and HT29-Snail1-HA cells. (**a**) Representative phase contrast images of LS174T-ctrl cells treated with 0.1 μg ml^{-1} Dox and DMSO, 50 nM LDN193189 (LDN), or 100 ng ml^{-1} Noggin (NOG) for 72 h as indicated. Scale bar: 100 μm. (**b**) Spheroid invasion assay of LS174T-ctrl cells treated with 0.1 μg ml^{-1} Dox and DMSO, 50 nM LDN193189 (LDN), or 100 ng ml^{-1} Noggin (NOG) for 96 h as indicated. One representative spheroid is shown for each condition. Scale bar: 200 μm. (**c**) Representative phase contrast images of LS174T-ctrl and LS174T-Snail1-HA cells treated with 0.1 μg ml^{-1} Dox and DMSO, 50 nM LDN193189 (LDN), or 100 ng ml^{-1} Noggin (NOG) for 144 h as indicated. Scale bar: 100 μm.(**d**) Representative phase contrast images of HT29-ctrl and HT29-Snail1-HA cells (clones 4F5 and 3C2) treated with 1 μg ml^{-1} Dox and DMSO, 50 nM LDN193189 (LDN), or 100 ng ml^{-1} Noggin (NOG) for 72 h as indicated. Scale bar: 100 μm.

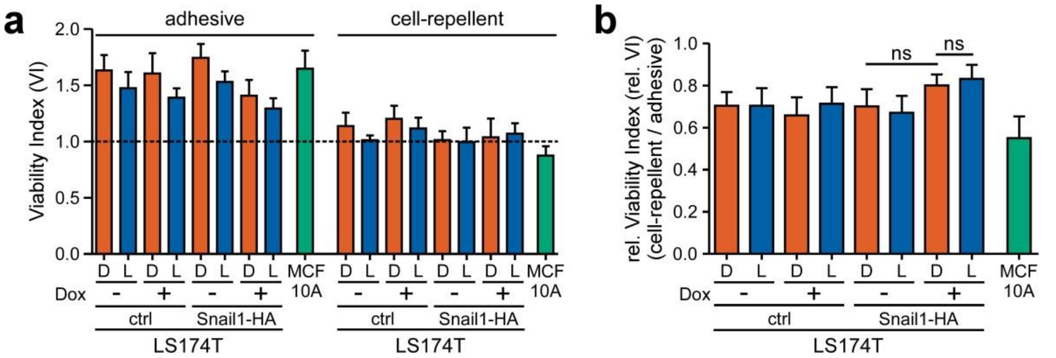

Figure S3: Anoikis resistance of LS174T-ctrl and LS174T-Snail1-HA cells with and without BMPi. (**a**) As a measure of anoikis resistance, the Viability Index (VI) was calculated by dividing the CCK8 assay absorbance readouts at 96 h by those at 72 h. The horizontal dotted line marks a VI of 1.0 above which cells are considered anoikis-resistant. MCF10A cells were analyzed in parallel as example for anoikis-sensitivity. Shown is the mean + SEM; n≥3. D: DMSO; L: LDN193189; Dox: doxycycline. (**b**) Relative Viability Indices (rel. VI) that were calculated by division of the obtained VIs in cell-repellent conditions by those in adhesive conditions (see (a)). MCF10A cells were analyzed in parallel as example for anoikis sensitivity. Shown is the mean+SEM; $n \geq 3$. ns: not significant. D: DMSO; L: LDN193189; Dox: doxycycline.

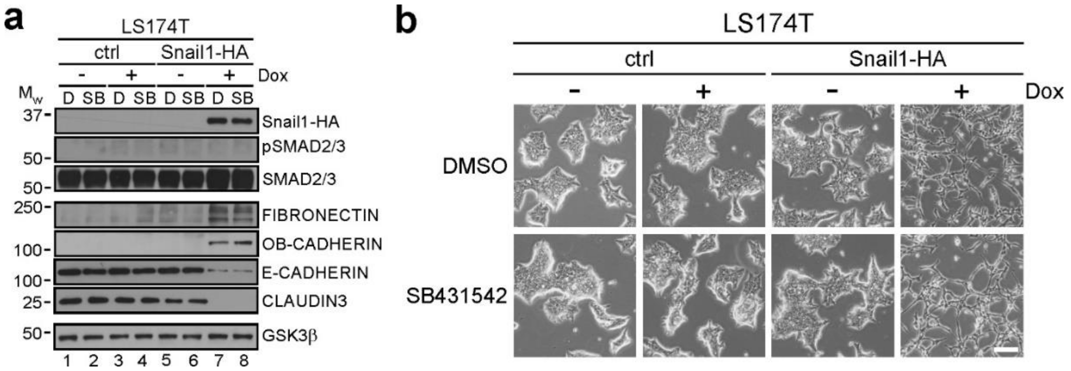

Figure S4: Chemical inhibition of TGFβR type I (ALK5) does not affect EMT in LS174T-Snail1-HA cells. (**a**) Western Blot analyses of whole-cell lysates. Names of detected proteins are indicated on the right. LS174T-ctrl and LS174T-Snail1-HA cells received 0.1 μg ml^{-1} Dox, DMSO (D), or 10μM SB431542 (SB) for 72 h as indicated. Positions of molecular weight (M_W) standards in kDa are given on the left. Detection of GSK3β was used as control for equal loading. As not all proteins could be analyzed on the same membrane, only one representative loading control is shown for reasons of simplicity. All corresponding loading controls for the images depicted can be found in Figure S9. (**b**) Representative phase contrast images of LS174T-ctrl and LS174T-Snail1-HA cells treated with 0.1 μg ml^{-1} Dox, DMSO or 10 μM SB431542 for 72 h as indicated. Scale bar: 100 μm.

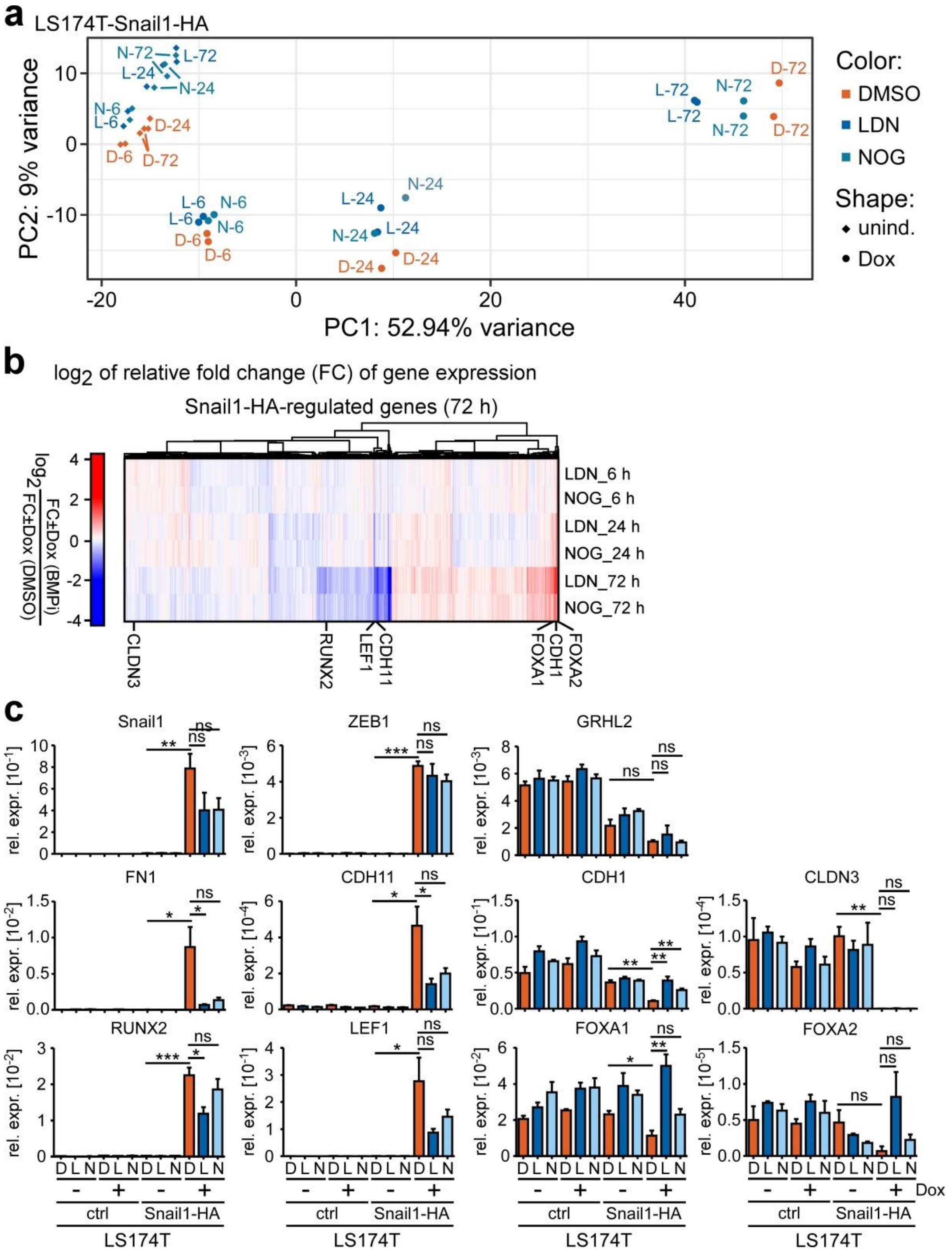

Figure S5: Transcriptome changes by BMP inhibition during SNAIL1-induced EMT. (**a**) Principal component analysis (PCA) of microarray-based transcriptomic analysis in LS174T-Snail1-HA cells. Cells were treated with 0.1 µg ml^{-1} Dox, 50 nM LDN193189 (L, LDN), 100 ng ml^{-1} Noggin (N, NOG) or DMSO (D). For each condition, two biological replicates were analyzed. (**b**) Heatmap showing the impact of BMP inhibitor treatment on the regulation of all genes regulated by Snail1-HA after 72 h of 0.1 µg ml^{-1} Dox administration. Plotted is the $\log_2$ of the relative fold change of gene expression under BMP inhibitor treatment. Relative fold change was calculated by dividing gene expression differences induced by Snail1-HA in the scenario of BMP inhibition by the differences evoked by Snail1-HA under control conditions. Inhibitors used and durations of treatment are indicated on the y-axis. Positions of genes of interest are given on the bottom. (**c**) qRT-PCR analyses of mRNA expression in LS174T-ctrl and LS174T-Snail1-HA cells. Where indicated, cells were treated with 0.1 µg ml^{-1} Dox, 50 nM LDN193189 (L), 100 ng ml^{-1} Noggin (N) or DMSO (D) for 72 h. Shown is the mean + SEM; $n = 3$. Rel. expr.: relative expression normalized to that of *GAPDH*. ns: not significant. *: $p < 0.05$, **: $p < 0.01$, ***: $p < 0.001$.

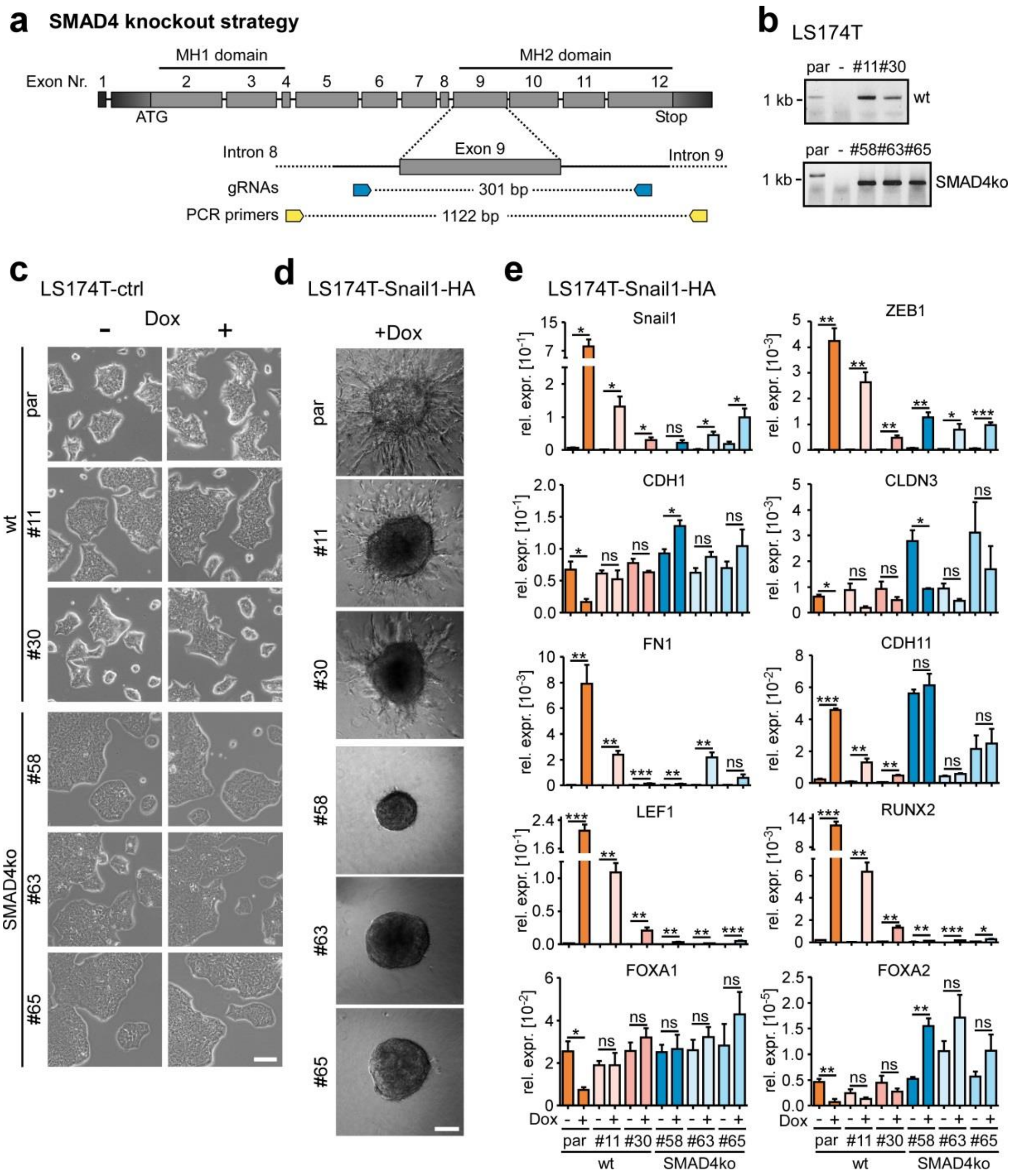

Figure S6: Knocking out *SMAD4* in LS174T cells using CRISPR/Cas9. (**a**) Schematic of knockout strategy for *SMAD4*. Shown is the gene structure of *SMAD4*. Introns and UTRs (dark grey) are not drawn to scale. Approximate locations of the two main functional domains of SMAD4 (MH1 and MH2) are shown on top. Blue arrows indicate the location of the two gRNAs used, producing a ≥301 bp deletion that contains Exon 9. Yellow arrows show the position of primers used for the PCR screening. (**b**) Agarose gel electrophoresis results obtained from PCR screening of the selected single cell clones. Parental (par) LS174T cells were used as positive control. The negative control (-) did not have any input-DNA. Clone numbers are given on top of the gels. The position of a length standard is indicated on the left. (**c**) Morphology of LS174T parental (par) cells and single cell clones reconstituted with an insert-free inducible expression vector. Cells were left untreated or received 0.1 µg ml^{-1} Dox for 72 h. Scale bar: 100 µm. (**d**) Spheroid invasion assay of LS174T- parental (par) cells and single cell clones reconstituted with an inducible expression vector for Snail1-HA. Cells were treated with 0.1 µg ml^{-1} Dox, for 192 h. One representative spheroid is shown for each condition. Scale bar: 200 µm. (**e**) qRT-PCR analyses of mRNA expression LS174T- parental (par) cells and single cell clones reconstituted with an inducible expression vector for Snail1-HA. Where indicated, cells received 0.1 µg ml^{-1} Dox for 72 h. Shown is the mean + SEM; $n = 3$. Rel. expr.: relative expression normalized to that of *GAPDH*. ns: not significant. *: $p < 0.05$, **: $p < 0.01$, ***: $p < 0.001$.

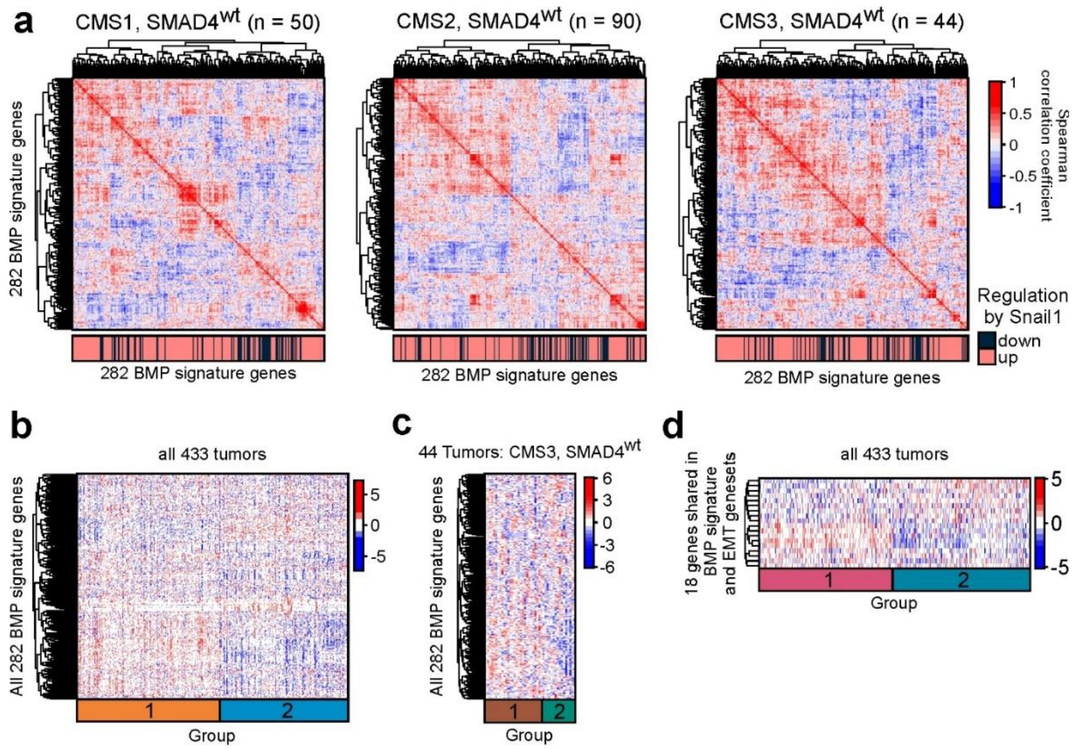

Figure S7: Expression of BMP signature genes in human tumor samples. (**a**) Correlation maps showing the mutual correlation of expression levels of all 282 BMP signature genes in transcriptomes of colorectal cancer (CRC) samples from TCGA. Only tumors without mutations in SMAD4 (SMAD4^{wt}) were considered and further stratified according to their consensus molecular subtype (CMS) classification. Genes were clustered by unsupervised hierarchical clustering. The color bar on the bottom of each plot indicates whether a gene is up- or downregulated by Snail1-HA in LS174T cells. (**b–d**) Heatmaps showing expression of BMP signature genes in human colorectal tumor samples from TCGA. The respective cancer samples used for the analysis are indicated on top, while the set of genes applied is shown on the left. Patients were separated in two groups as indicated on the bottom using unsupervised k-means clustering, with k = 2. Color code represents the row-wise Z-score count per million.

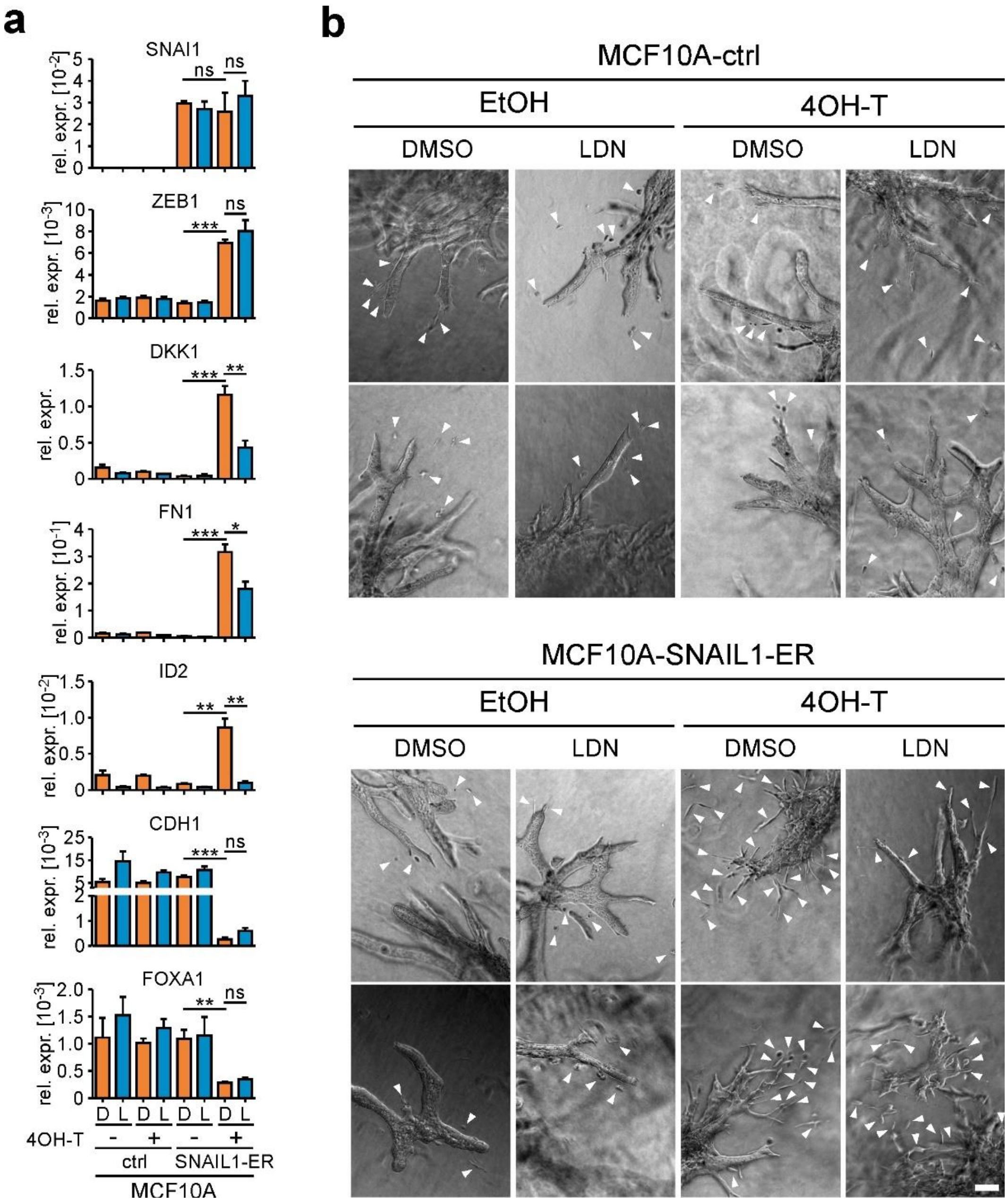

Figure S8: Contribution of BMP pathway activity to EMT in human mammary epithelial cells. (**a**) qRT-PCR analyses of mRNA expression in MCF10A-ctrl and MCF10A-SNAIL1-ER cells. Cells were treated with ethanol (-) or 100 nM 4-hydroxytamoxifen (4OH-T) and DMSO (D) or 50 nM LDN193189 (L) for 10 days as indicated. Shown is the mean + SEM; n = 3. Rel. expr.: relative expression normalized to that of *GAPDH*. ns: not significant. *: $p < 0.05$, **: $p < 0.01$, ***: $p < 0.001$. (**b**) Spheroid invasion assay of MCF10A-ctrl and MCF10A-SNAIL1-ER cells treated with ethanol (EtOH) or 100 nM 4-hydroxytamoxifen (4OH-T) and DMSO or 50 nM LDN193189 (LDN) for 14 days as indicated. Two representative fields of view derived from different biological replicates are shown for each condition. White arrowheads mark invaded single cells that were considered for quantification (see Figure 6c). Scale bar: 100 μm.

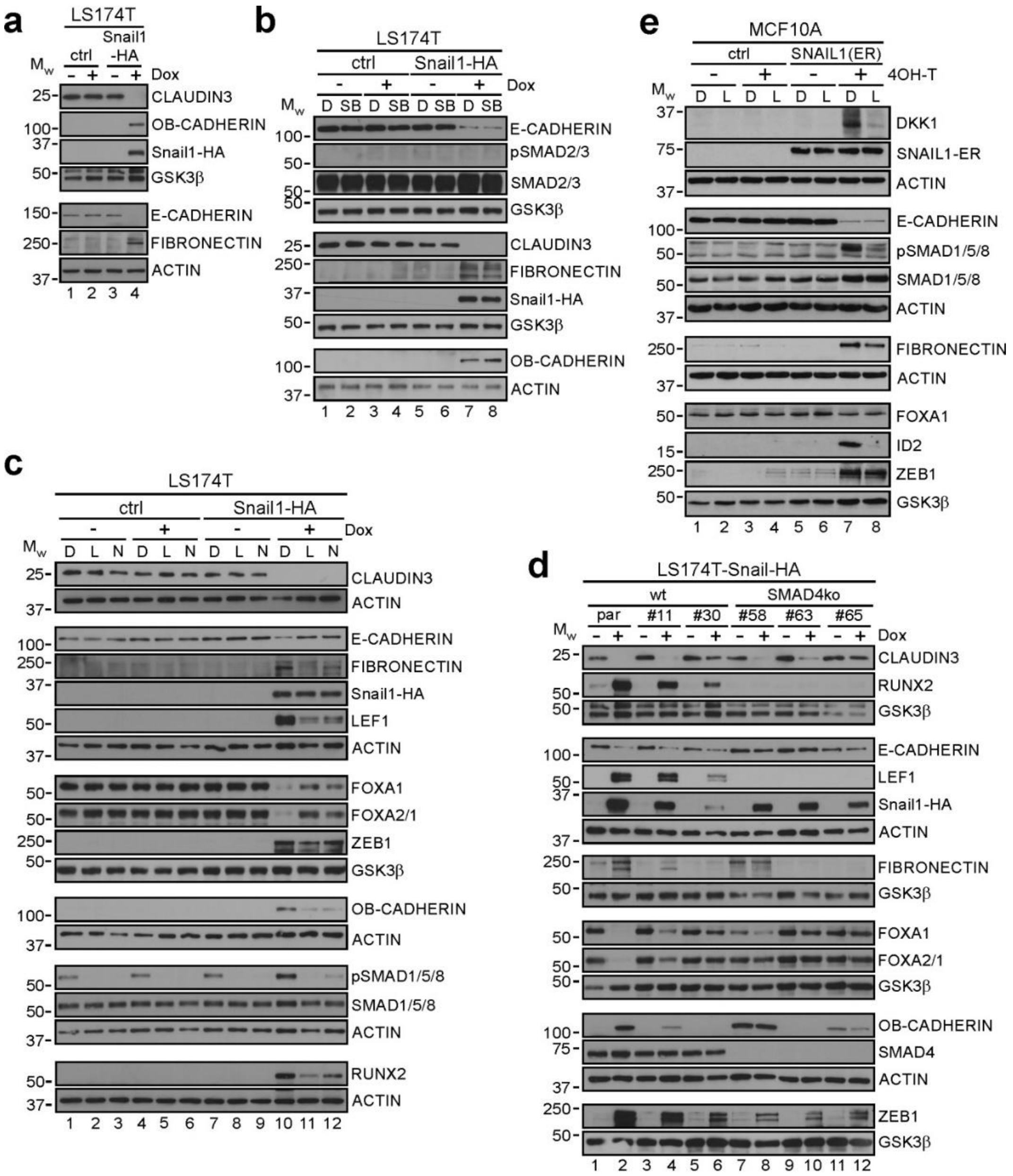

Figure S9: Compiled immunoblots from all figures including the corresponding loading controls for every detection ordered alphabetically. Detection of ACTIN and GSK3β was used as control for equal loading. Proteins were detected using whole-cell lysates, except for FOXA1, FOXA2, ID2 and ZEB1 for which nuclear extracts were used. Positions of molecular weight (Mw) standards in kDa are always given on the left. Images relate to (**a**) Figure 1c (**b**) Figure S4a (**c**) Figure 3e (**d**) Figure 4e (**e**) Figure 6b.

related to Figures 1c, S9a:

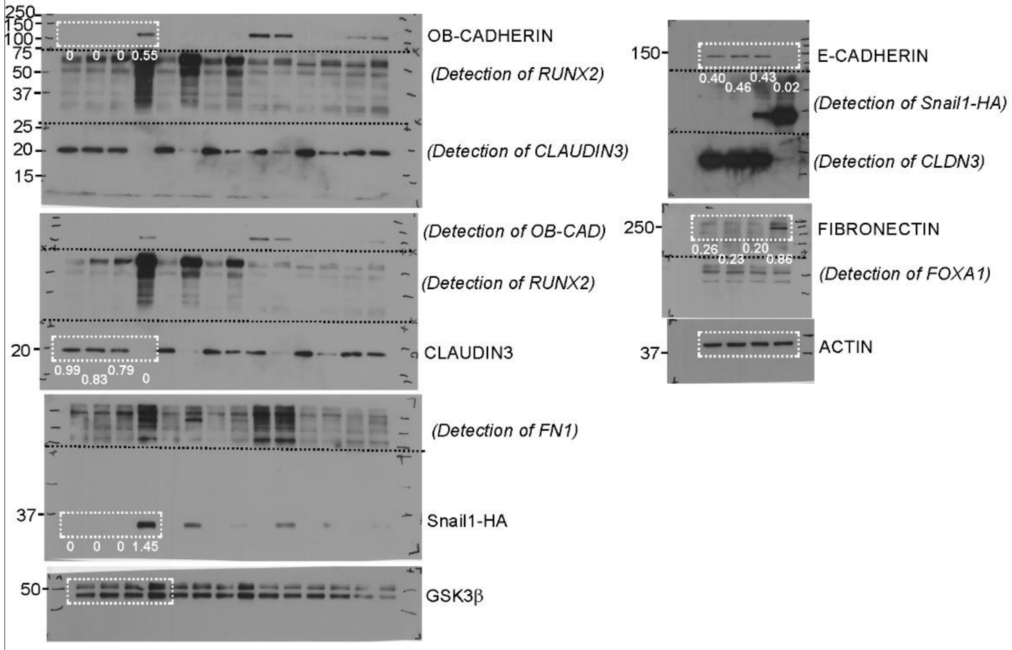

related to Figure S1a:

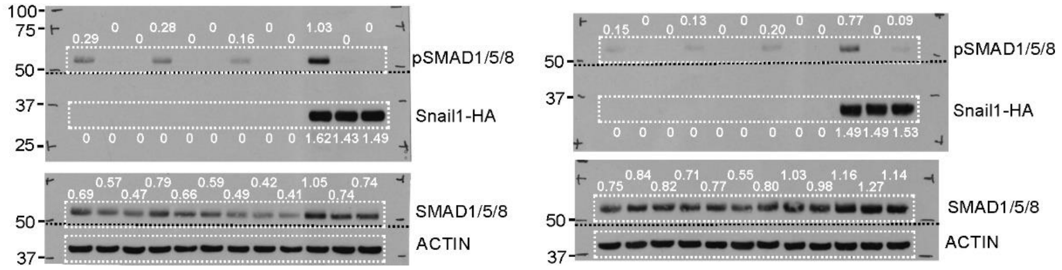

related to Figure S1b:

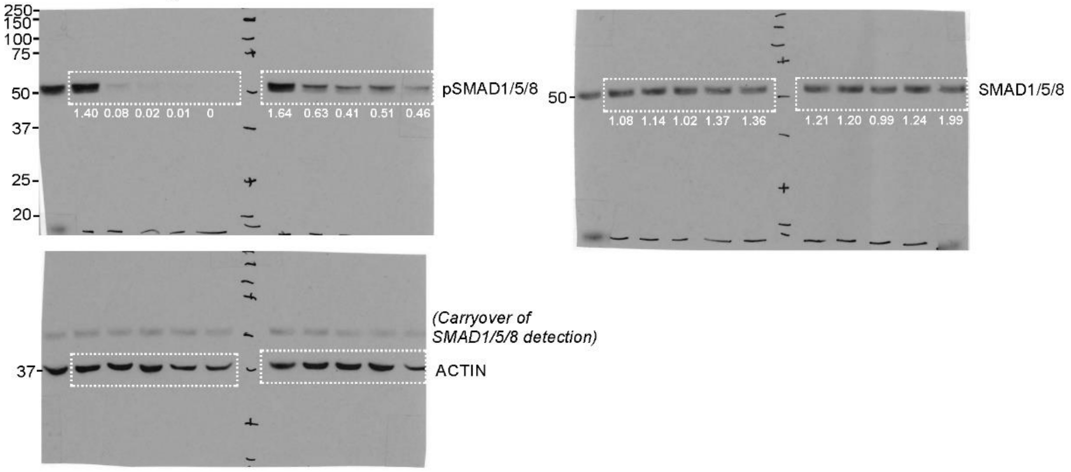

related to Figures 2b, S1d:

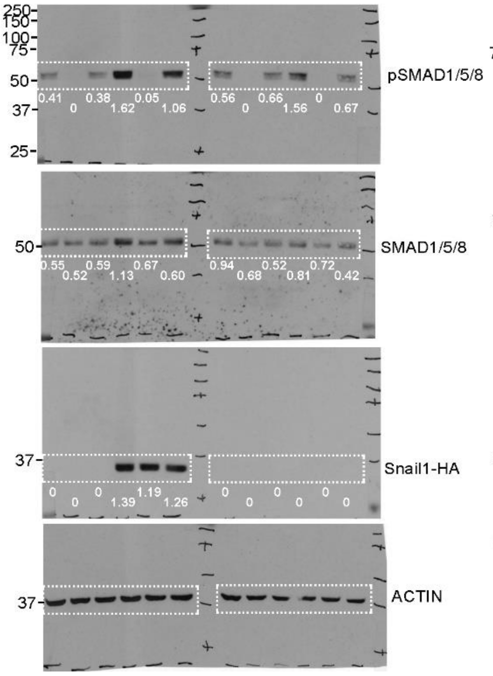

related to Figures 2c, S1d:

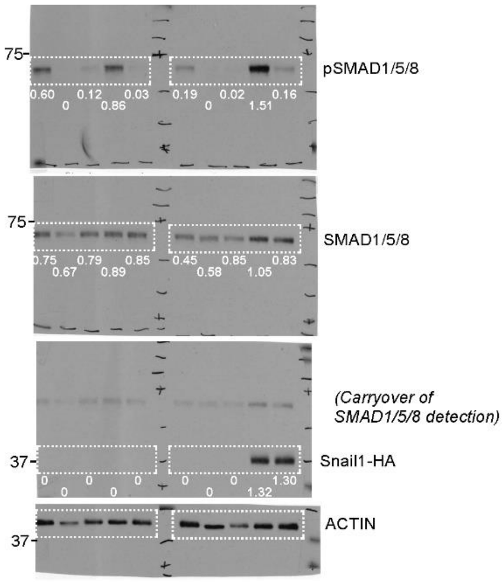

related to Figures S4a, S9b:

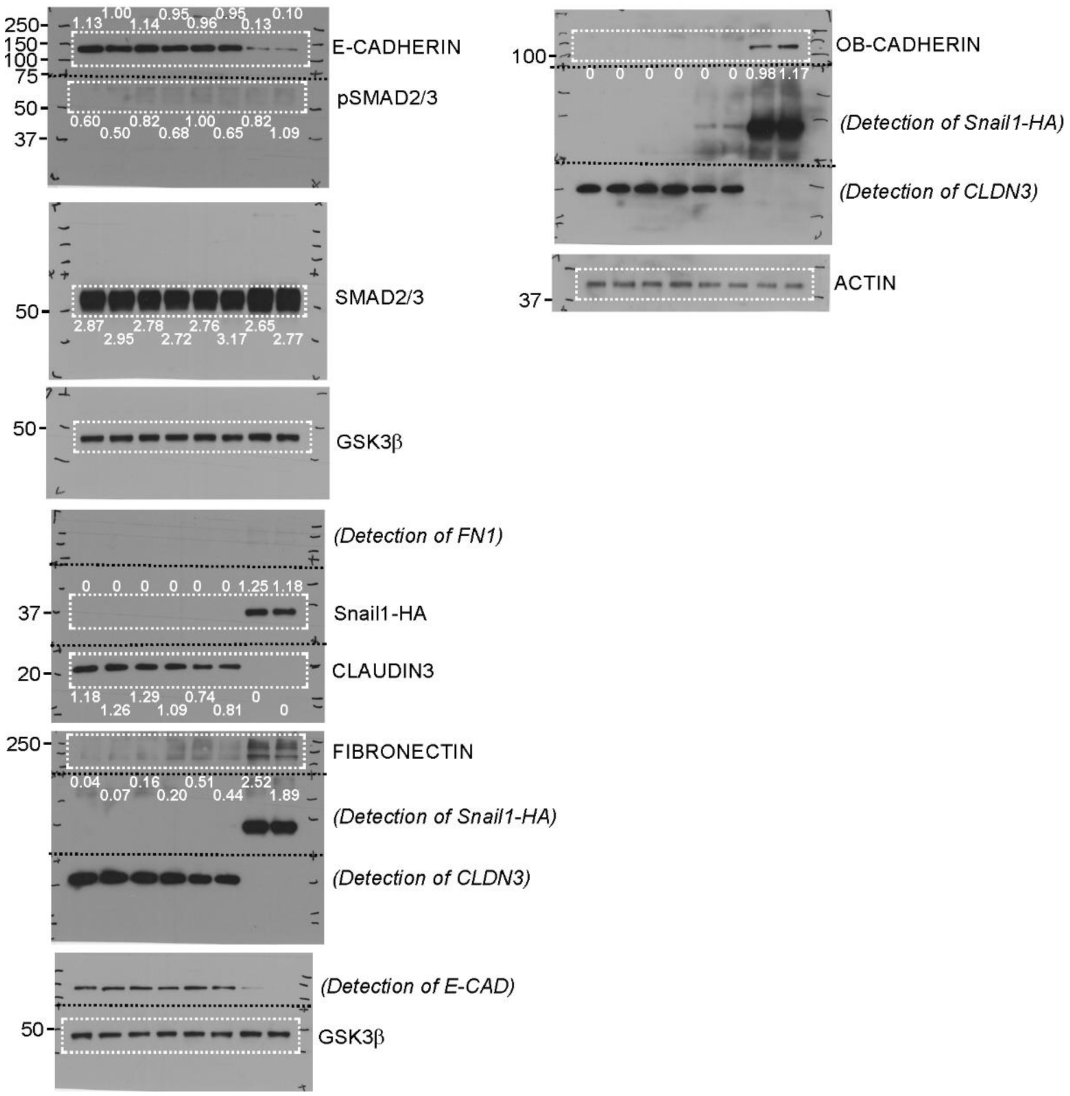

related to Figures 3e, S9c:

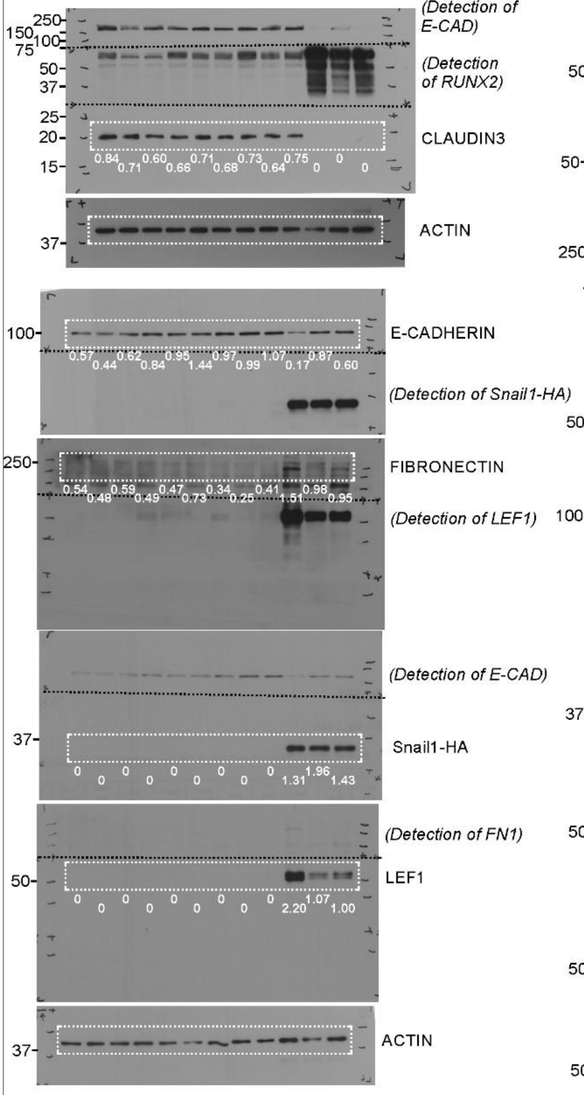

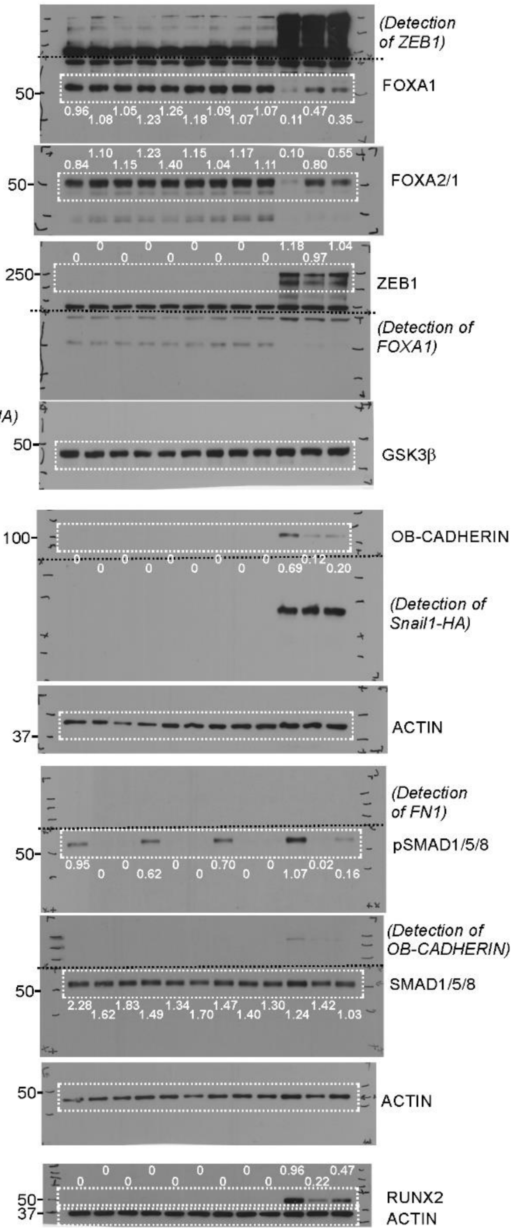

related to Figure 4a:

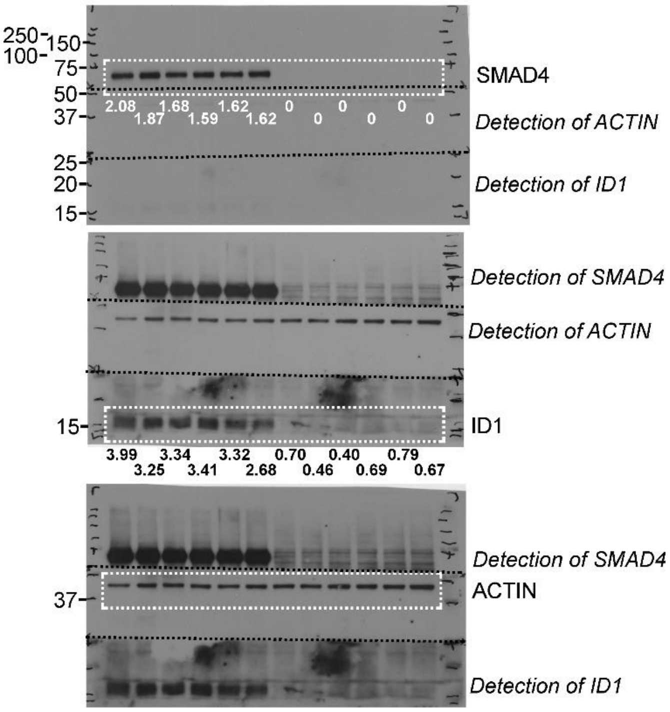

related to Figures 4e, S9d:

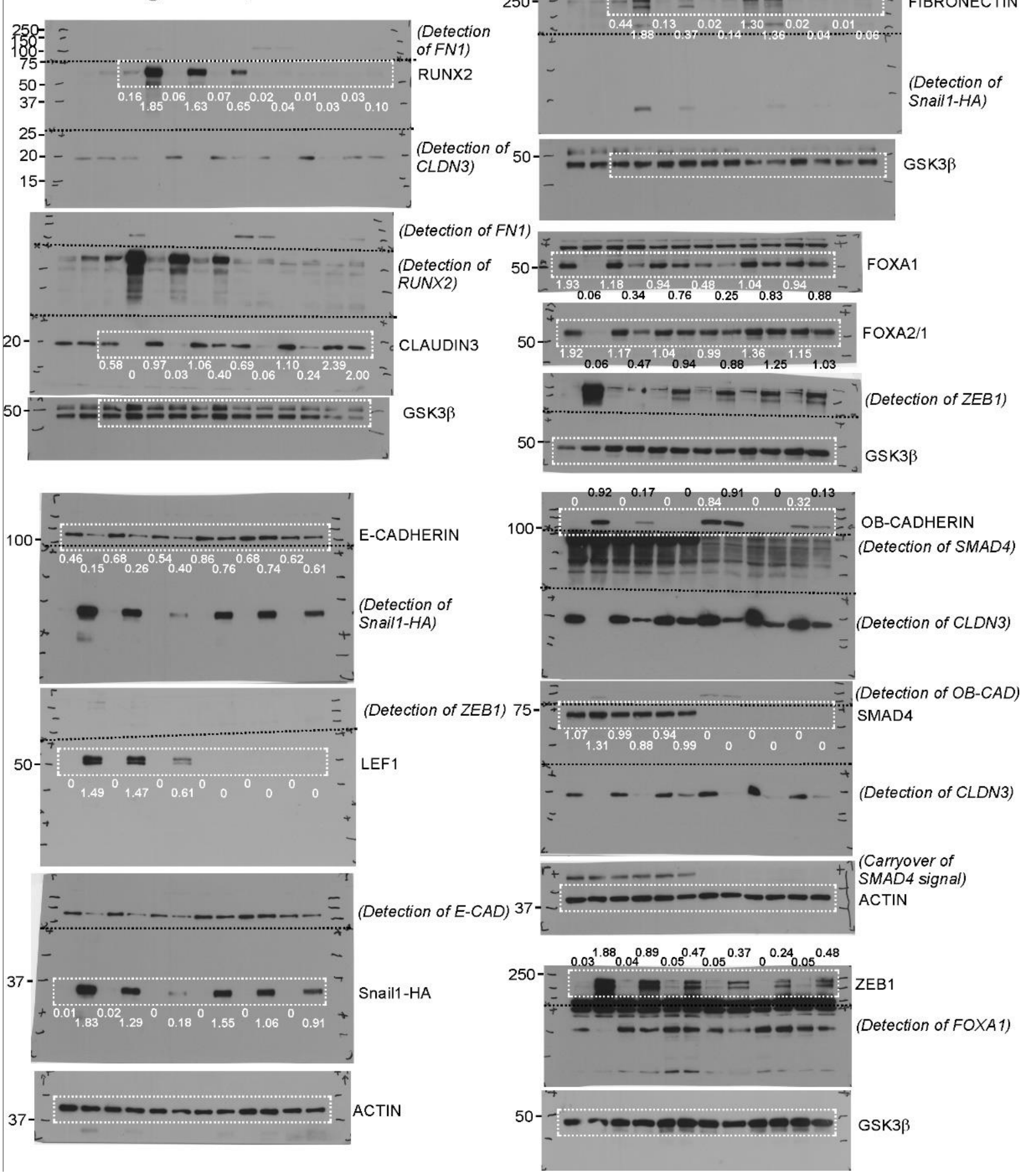

related to Figures 6b, S9e:

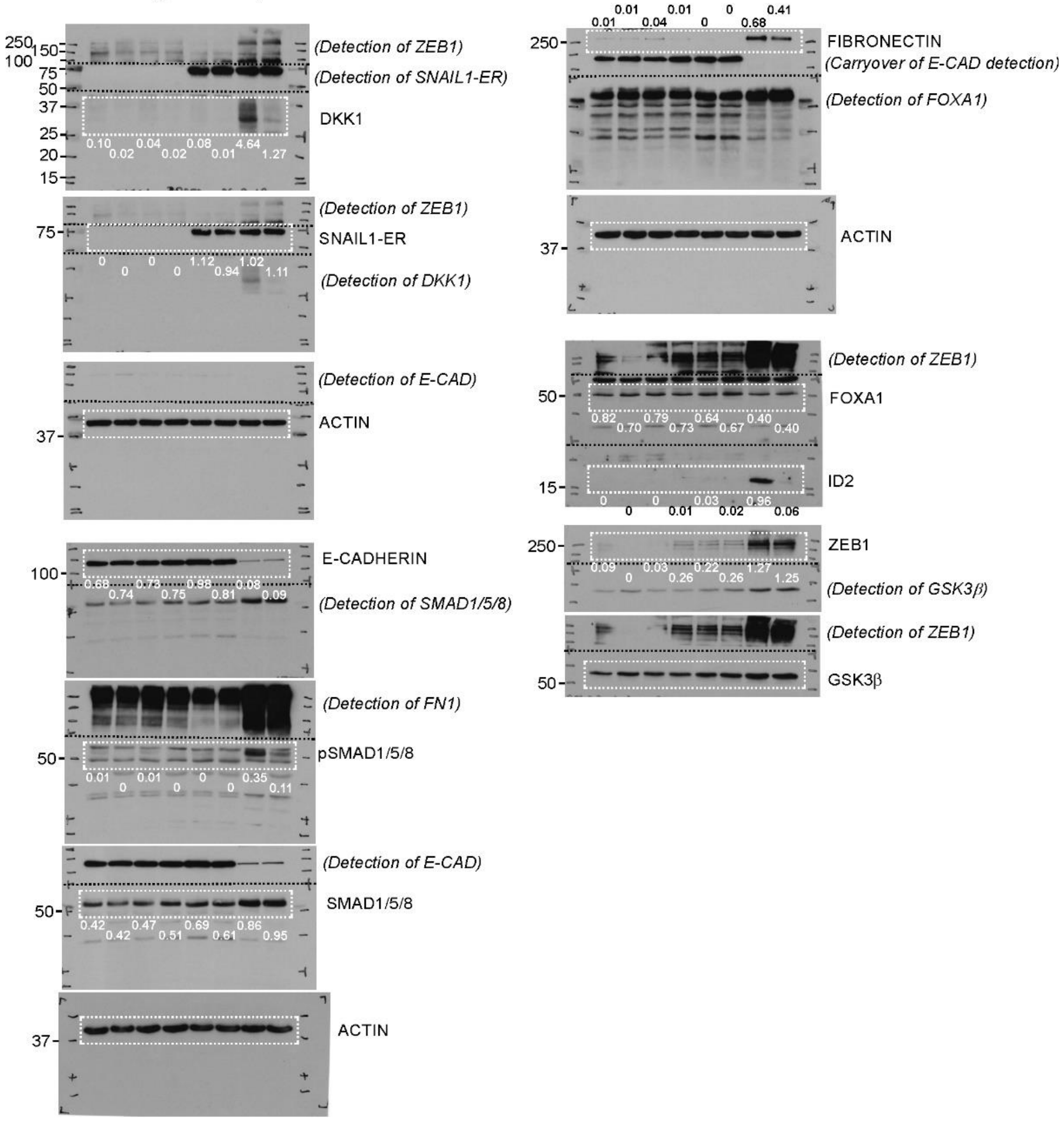

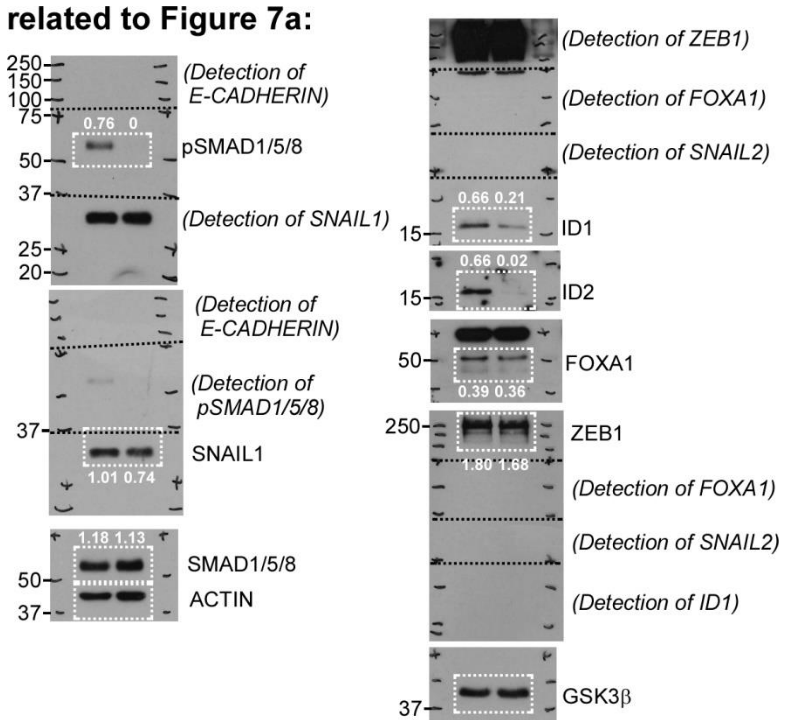

Figure S10: Compilation of uncropped immunoblots for all figures including densitometry readings. The corresponding figures where the cropped versions are shown are indicated on top of the blots. Dotted white rectangles highlight the sections that were used for figure preparation with the detected proteins given on the right. In cases were membranes were cut to enable simultaneous detection of different epitopes, dotted black lines indicate the introduced cuts. Densitometry values of bands relative to the intensity of the respective loading control band are indicated. The position of one molecular weight standard [kDa] per blot is given on the left. Simultaneous detections carried out on the same membrane that were not used for figure preparation as well as carryovers of signal from previous detections on the membranes are indicated in italics.

© 2020 by the authors. Licensee MDPI, Basel, Switzerland. This article is an open access article distributed under the terms and conditions of the Creative Commons Attribution (CC BY) license (http://creativecommons.org/licenses/by/4.0/).
